# Right ventricular kinetic energy: 4D flow MRI analysis of healthy volunteers and repaired Tetralogy of Fallot

**DOI:** 10.1186/1532-429X-16-S1-O46

**Published:** 2014-01-16

**Authors:** Daniel Jeong, Alejandro Roldán-Alzate, Christopher J Francois

**Affiliations:** 1 Department of Radiology, University of Wisconsin, Madison, Wisconsin, USA; 2 Department of Medical Physics, University of Wisconsin, Madison, Wisconsin, USA

## Background

Repaired Tetralogy of Fallot (rTOF) is currently monitored with Cardiovascular MR (CMR) to measure right ventricle (RV) volumes and function. However, these indices do not always reflect performance status or predict success following repair of pulmonary insufficiency. RV kinetic energy (KERV) may provide a more robust measure of RV efficiency than RV end diastolic volume (EDV) index and function. The aim of this study was to demonstrate the feasibility of calculating KERV with 4D Flow MRI and assess differences between rTOF and healthy volunteers.

## Methods

Ten subjects with rTOF and 9 healthy volunteers were scanned according to an IRB approved protocol. 4D flow MRI (Phase Contrast Vastly Undersampled Isotropic Projection Reconstruction PCVIPR) was performed on clinical MRI scanners (GE Healthcare, Waukesha, WI) after administration of gadolinium based contrast agents. PC VIPR parameters: FOV = 260-320 mm3, isotropic 1.3 mm spatial resolution, TR/TE = 8.8-10.9/2.8-3.7 ms (1.5T), and 6.2-3.7/2.0-2.2 ms (3.0T), Venc = 40-400 cm/s, scan time: 9-17 minutes using respiratory and retrospective ECG gating. Post processing was done using Mimics (Materialise, Leuven, Belgium) and MatLab (The MathWorks, Natick, MA) to measure KERV and main pulmonary artery flow (QMPA) throughout the cardiac cycle. The KE of a voxel of blood was calculated from 1/2 mv^2, where the mass (m) represents the voxel volume multiplied by the density of blood (1.025 g/ml) and the velocity (v) of each voxel was determined from 4D flow MRI. KERV was determined from the sum of the KE of the voxels within the segmented RV volume at each phase of the cardiac cycle. Differences in peak systolic and diastolic KERV and the QMPA/KERV ratio, an indicator of RV-MPA coupling, between groups were assessed using the Wilcoxon rank-sum test.

## Results

**Figure 1:**
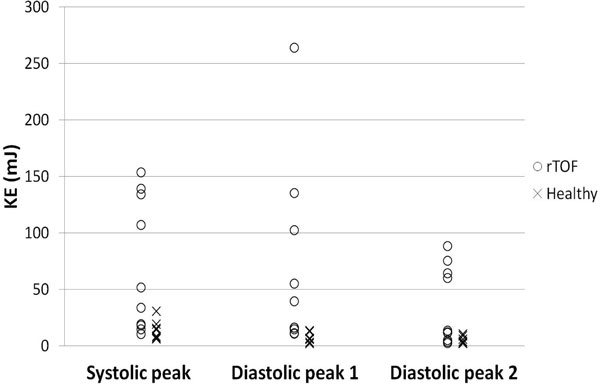
**RV KE comparison between rTOF (O) and healthy volunteers (X)**. Differences in peak systolic (p = 0.0076) and diastolic (p = 0.0003 peak 1, p = 0.013 peak 2) RV KE are statistically significant.

**Figure 2:**
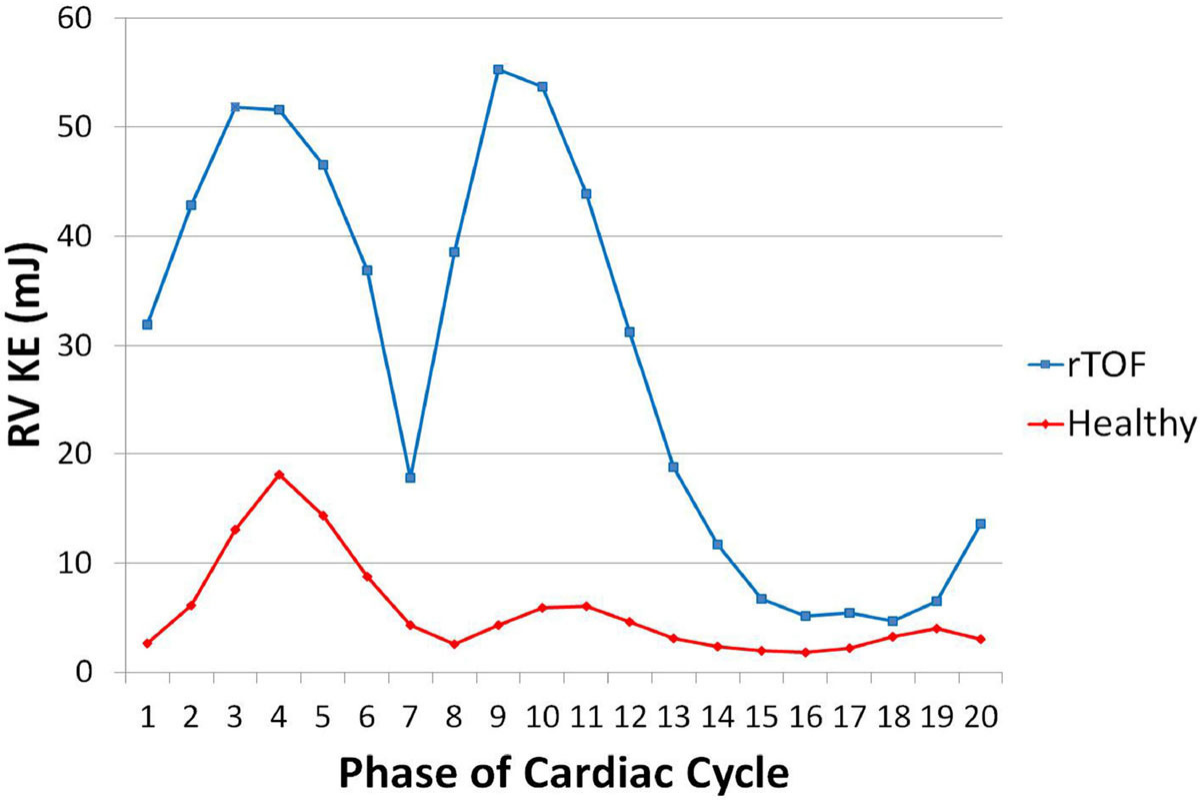
**RV KE throughout cardiac cycle for a representative subject from rTOF and healthy volunteer groups**. Three distinct KE peaks were observed in all subjects.

Three distinct KE peaks were observed in all subjects. Peak systolic (p = 0.0076) and diastolic (p = 0.0003 peak 1, p = 0.013 peak 2) were higher in rTOF than in healthy volunteers (Figures 1,2). The QMPA/KERV ratio was lower in rTOF (0.92 +/- 0.68 ml/cycle*mJ) than in healthy volunteers (6.92 +/- 4.16, p = 0.0002).

## Conclusions

Time-resolved KERV was measured in rTOF and healthy volunteers using 4D flow MRI. KERV was higher and QMPA/KERV ratio was lower in rTOF than in healthy volunteers, indicative of greater inefficiency in RV function to generate the same cardiac output. Future studies are needed to determine if changes in RV KE provide earlier evidence of RV dysfunction and need for reintervention than standard CMR measurements.

## Funding

NIH grant R01HL07226. GE Healthcare for research support.

